# Effects of Heavy Metals in Lake Water and Sediments on Bottom Invertebrates Inhabiting the Brackish Coastal Lake Łebsko on the Southern Baltic Coast

**DOI:** 10.3390/ijerph17186848

**Published:** 2020-09-19

**Authors:** Natalia Mrozińska, Martyna Bąkowska

**Affiliations:** Department of Hydrobiology, Kazimierz Wielki University, 10 Al. Powstańców Wielkopolskich Str., 85-090 Bydgoszcz, Poland; bakowska.martyna@ukw.edu.pl

**Keywords:** heavy metals, macroinvertebrates, coastal lake, hydrological connectivity, water and sediments

## Abstract

Lake Łebsko is the largest and most productive coastal lake of the southern Baltic Sea to which it is permanently connected. The shoreline is well-developed, and the lake is divided into three parts: eastern, central, and western. Seawater intrusion affects most strongly the eastern part, where the Łeba River connects it with the sea. Samples of water and sediments were collected in 2014–2015. In the same places and time interval, bottom fauna was collected to determine the influence of environmental predictors on its qualitative-quantitative structure. Metals Cr (chromium), Pb (lead), Ni (nickel), Cu (copper), and Al (aluminium) in the samples were analyzed using inductively coupled plasma optical emission spectrometry. Most of the analyzed physicochemical variables of water were significantly higher in the eastern part: conductivity, salinity, sulfates (*p* < 0.0001) and chlorides (*p* = 0.01). Metal concentrations in water did not differ significantly between the lake parts, but in sediments they were generally higher in the western part. During the study, we detected significant changes in descriptors and abundance of the major groups of benthic fauna (*Oligochaeta* and *Diptera*), mostly between the eastern and western parts. BIO-ENV analysis showed that the benthic community of Lake Łebsko is shaped primarily by physicochemical variables of water (42% of the variance), linked with intrusion of seawater. Secondarily, the structure of the benthic community is affected by the amounts of heavy metals in sediments (31%) and water (12%). The findings can help us improve the principles of management of coastal lakes, including modification of hydrological conditions.

## 1. Introduction

Coastal lagoons are ecosystem hotspots, which because of the ongoing dynamic changes at the interface between land and sea are important indicators of proper functioning of the hydrosphere [1]. Their progressive pollution, especially with heavy metals, is now a global problem [2]. Heavy metals accumulate in food chains, posing serious threats to public health [3] and the environment. Some heavy metals, such as Cr (chromium), Ni (nickel), Cu (copper), and Al (aluminium), are necessary in trace amounts for the fauna and flora and are recommended as dietary supplements [4]. Some others are treated as highly toxic, e.g., aluminium, which should not exceed the upper permissible limits in aquatic ecosystems; otherwise, they are treated as harmful. The limits differ depending on water type and application, country and the synergistic negative effects of pollution on living organisms [5]. Increased concentrations of heavy metals in water may lead to their toxic influence on fauna and flora in the pelagic zone. Simultaneously, a stronger impact is observed in the benthic zone [6], where organisms interact with metals accumulated in the sediments colonized by them. Heavy metal ions, hardly soluble in water, are leached from the catchment area and transported to reservoirs, lakes, seas, and oceans. In watercourses, some metals are also deposited in sediments, although kinetic forces of individual molecules do not facilitate these processes [7]. As a result, sediments in coastal zones of seas and oceans are believed to be areas of increased accumulation of all water pollution, including trace metals [8]. This problem also applies to the southern Baltic coastline, where intermittently closed and open lakes and lagoons (ICOLLs) are recipients of water from rivers draining areas of several thousand km^2^ each (e.g., the Łeba, Łupawa, Rega). Thus, research on these ecosystems makes it possible to assess human impact, including heavy metal accumulation, which can be toxic.

**Figure 1:**
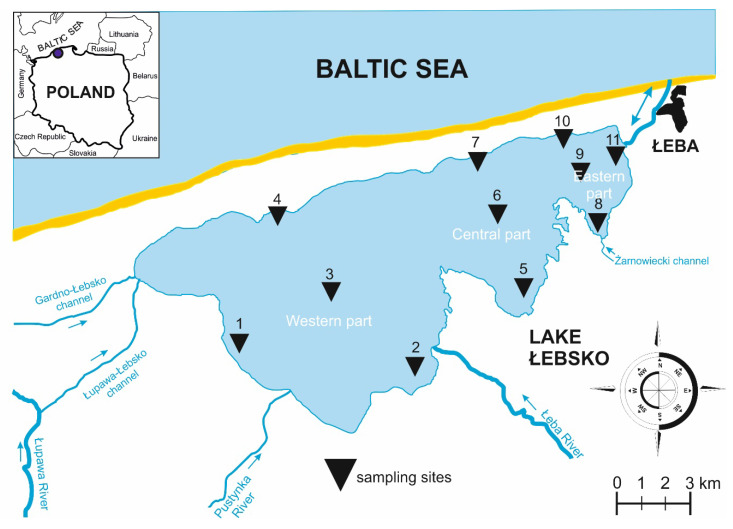
Map of sampling sites of Lake Łebsko, Poland, in 2014–2015.

Heavy metal circulation between water and sediments in shallow coastal lakes is relatively intensive, as wind causes resuspension of sediments and release of various elements into water. Additionally, it is regulated by ambient physicochemical conditions, such as pH, salinity, redox potential or the rate of organic matter decomposition. In transitional lakes, where fresh and seawater is mixed, these variables are treated as crucial [9]. In Lake Łebsko, located in the coastal zone of the southern Baltic Sea (Figure 1), intrusion of seawater often takes place, so the lake is classified as brackish. This affects the intensity of physicochemical and biological processes in open water and bottom sediments [10].

The lake is located within the Słowiński National Park, protected by the Ramsar Convention. It is connected with the Baltic Sea by the Łeba River mouth. Although it is the largest Baltic coastal lake, its area is gradually decreasing. Over the last century, the spread of emergent vegetation has caused a retreat of the shoreline at a rate of 0.3–3.0 m·year^−1^, so that the lake area has been reduced by some 104 ha. Moreover, about 67% of lake area is less than 100 cm deep [11]. Heavy metals accumulate in lake water and sediments, affecting the living conditions of benthic fauna, and can be regarded as sensitive predictors for pollution monitoring. The major anthropogenic sources of metals are: industrial waste, oil spills, and agricultural runoff, directed to the lake by drainage systems. Its tributaries feed the southern and western parts of the lake and carry agricultural pollution from point and areal sources. In the north-eastern part of the lake, the terminal section of the Łeba River is located, playing also the role of a seaport canal in the town of Łeba and the way of seawater intrusion. In the south-western part, the lake is fed by the Gardno-Łebsko Canal, connecting both lakes, and the Łupawa-Łebsko Canal, which is a by-pass linking valleys of two rivers of Polish Pomerania: the Łupawa and Łeba. As a result, Lake Łebsko is a sedimentation tank for pollution transported from an area of more than 2700 km^2^ [12].

A significant environmental predictor affecting the functioning of ICOLLs is their hydrological connectivity with the sea, which determines the functioning of individual components in these unique aquatic ecosystems [11,13]. More and more studies are conducted at the border between land and sea, but coastal lakes, because of their complicated hydrological systems, appear to be relatively poorly-studied aquatic ecosystems. The chemical composition of water, linked with the possible intrusion of seawater, causes a high variation in living conditions, is a stressor for many invertebrates [6,14] and affects geochemical changes in the biotope [15]. In lakes with the possible intrusion of seawater, a small salinity gradient is observed, conditioned by the specificity of these lakes. There are mathematical models for explanation of data, enabling water quality evaluation using unambiguous terms (e.g., excellent, good, poor). To a large extent they make use of ecological tolerance of organisms living in various zones of aquatic ecosystems. Increased concentrations of metals determine their behavior, which is particularly conspicuous in the case of benthic fauna, as trace elements are accumulated at the bottom. Thus ecotoxicological studies should provide information on potential routes of transmission of toxic metals from the level of bottom sediments and water to flora, fauna, and *Homo sapiens*, as a top predator in the food web [16].

The major aim of this study was to assess the influence of heavy metal pollution (Al, Ni, Pb, Cu, Cr) of both water and sediments of the brackish costal Lake Łebsko on the structure of benthic invertebrate communities. For this purpose, we quantified the importance of environmental predictors (physicochemical variables of water, metal concentrations in water and sediments) in shaping biotic components. We compared three parts of the lake, which differ in salinity level.

## 2. Materials and Methods

### 2.1. Description of the Study Area

Lake Łebsko is a brackish coastal lake separated from the Baltic Sea by a sandbar, which is about 15 km long [17]. In respect of surface area, it is the largest coastal lake on the southern coasts of the Baltic Sea and 3rd largest lake in Poland. The sandbar separating the lake from the sea is crossed by the Łeba River, which is a hydrological connection between both ecosystems. The water body is within the catchment area of the Łeba (its main tributary). Łebsko is also fed by smaller tributaries: the Pustynka River, Żarnowski Canal, Łupawa-Łebsko Canal, and Gardno-Łebsko Canal. The northern shoreline is covered mostly by forest, while the western, southern, and eastern shores are dominated by fens and other wetlands, occupying extensive areas. The mean depth of Łebsko is only 1.6 m, but due to its large surface area its volume is about 117.5 million m^3^ (Table 1).

The bottom is relatively flat. This results from strong wave action, causing frequent mixing of the whole water volume (polymictic lake), which flattens the bottom. Only in the axial part of the lake some deeper places are located. The lake bottom is a cryptodepression. The whole Lake Łebsko is located within the Słowiński National Park, accounting for as much as 21.8% of its total area [18]. The shoreline is highly varied, dividing the lake into three parts: western (largest), central, and eastern (smallest). The sampling sites (Figure 1) were selected to show the distribution of invertebrates and heavy metals in various parts of the lake, due to mixing of fresh water with seawater.

### 2.2. Sample Collection

Samples for chemical and biological analyses were taken in 2014–2015, from each sampling site three times a year: in spring, summer, and autumn. In field conditions we measured temperature, pH, dissolved oxygen (%), conductivity, salinity, and chlorides, using AquaProbe 7000 (AquaRead, Kent, UK). For laboratory analyses, water samples were collected from the depth of 0.5 m to 1 dm^3^ polyethylene containers, whereas sediments and invertebrates, sampled using an Ekman bottom grab with a catching area of 225 cm^2^, were transferred to 1 dm^3^ glass containers. Water samples were filtered through ash-free filter paper and afterwards kept at −15 °C, like the collected sediments. In the case of biological samples, the procedure was repeated three times, and the material was passed through a sieve (mesh size 0.5 mm). Biological material was preserved in situ with 4% formalin [6,14].

### 2.3. Laboratory Procedures

Water samples were analyzed in the laboratory within 24 h after collection. Their ion composition (Na^+^, K^+^, Ca^2+^, Mg^2+^, and SO_4_^2−^), indicating the level of seawater intrusion into the lake, was assessed using an ion chromatograph (881 Compact IC Pro, Metrohm, Herisau, Switzerland). Before analyses, the samples were filtered with the use of sterile filters (0.20 μm), and next examined using columns Metrosep C4 250/4.0 and Metrosep A Supp 5 250/4.0 as well as Metrosep C4 Guard/4.0 and Metrosep A Supp 4/5 Pre-column Guard 4.0, respectively.

The sediments in the laboratory were rewarmed, dried at 65 °C, and next ground to a fine homogeneous powder. For metal analyses, sediment samples (1 g) were digested with 15 mL of concentrated HNO_3_ and 5 mL of H_2_O_2_, and next heated for 2 h with a heating mantle. After mineralization, water sample was diluted to 50 cm^3^, using deionized, highly purified water which was used in blank tests.

Water samples for metal analyses were evaporated to 20–25 cm^3^, and next mineralized, using an analogous procedure described above for sediments. All the used reagents were of analytical grade. For preparation of the reference materials and samples, we used 65% HNO_3_ (Merck, Darmdtadt Germany) and 30% hydrogen peroxide (Sigma Aldrich, Steinheim, Germany).

To assay Cr, Pb, Al, Ni, and Cu, we used an Agilent 5100 inductively coupled plasma optical emission spectrometer (ICP-OES, Agilent, Santa Clara, CA, USA). Synchronous vertical dual view (SVDV) of plasma was achieved using the technology of dichroic spectral combiner (DSC), which allows simultaneous analysis of the axial and radial view. The chemical analysis was repeated three times to obtain reliable results with minimized measurement errors. The results were averaged only if the differences between the three results were lower than 5%. In other cases, the whole procedure was repeated. Each time, the same conditions were used:

(1) radio frequency (RF) power: 1.2 kW;
(2) gas flow through a nebulizer: 0.7 L·min^−1^;
(3) auxiliary gas flow: 1.0 L·min^−1^;
(4) plasma gas flow: 12.0 L·min^−1^;
(5) Charge Coupled Device (CCD) temperature: −40 °C;
(6) viewing height for radial plasma observation: 8 mm for 5 s.

For ICP-OES, we used commercial analytical Inductively Coupled Plasma (ICP) standards (Romil Ltd., Cambridge, UK). Detection limits, determined as 3-σ criteria, were estimated at 0.001 mg·L^−1^ and 0.01 mg·kg^−1^ dry weight (DW) for all the studied elements. The uncertainty of the whole analytical procedure (including sample preparation) reached 20%. Standard reference materials were: CRM S−1—loess soil; CRM NCSDC (73,349)—shrub twigs and leaves; CRM 2709—soil. To control the quality of the analysis, we used CRM 405 and CRM 667—estuary sediments. The recovery rate of 80–120% was acceptable for all the elements.

The assays of heavy metals in sediments allowed us to calculate the contamination factor (CF), which is the ratio of element concentration to the value of the geochemical background [19,20]:

(1) $$\mathrm{CF}\;=\;\frac{C_{n}}{B_{n}}$$

where *C_n_* is the concentration of element n in the sediment, and *B_n_* is the geochemical background of element n). CF values were calculated for each sampling site and lake part. In this study, the background concentration values are mean values determined by [8] for lakes on the southern coasts of the Baltic Sea. On the basis of CF, we classified pollution level as low (CF: <1), moderate (CF: 1–3), high (CF: 3–6), and very high (CF: >6) [19,20].

To assess heavy metal pollution of the sediments for individual elements and lake parts, we calculated the pollution load index (PLI) [21,22]. It is the *n^th^* root of the product of all (*n*) CFs [23]:

(2) $$\mathrm{PLI}\;=\;\sqrt[{n}]{{\mathrm{CF}}_{1}*\;{\mathrm{CF}}_{2}*{\mathrm{CF}}_{3}*\ldots *{\mathrm{CF}}_{n}}$$

where *n* is the number of analyzed trace elements. Since PLI takes into account all the CFs jointly, it reflects the overall level of heavy metal pollution of the sediments. PLI values higher than 1 indicate the presence of pollution, while values lower than 1 indicate the absence of pollution [24].

Biological material in the laboratory was sorted under a SZX16 stereo microscope equipped with the CellSens software for image analysis (Olympus, Tokyo, Japan). All invertebrates were identified to the lowest possible taxonomic level and counted. On the basis of the collected data, we calculated the basic community structure indices: abundance (ind. m^−2^), *α*-diversity index based on Shannon *H*′ index (log_2_), evenness index (*J′ = H′*/log [taxon number]), and *β*-diversity based on Whittaker index.

### 2.4. Statistical Analysis

To assess the overall differences in heavy metal concentrations in lake water and sediments and the structure of benthic invertebrate communities, we compared three parts of the lake (eastern, central, and western) in relation to physicochemical conditions of water. We performed one-way analysis of variance (ANOVA) with Kruskal–Wallis test (*K–W*) and the post hoc Dunn multiple comparison test, using GraphPadPrism 5.01 software (GraphPad, San Diego, CA, USA). At that stage, the data were tested for normality (Shapiro–Wilk test) and homoscedasticity (Levene test) and next log-transformed (x + 1) [25]. Procedures of correction of the level of type I errors in many test situations were performed (Tukey method). This method ensures a strong control of type I errors, is very conservative, as if comparisons are numerous, some real differences can be overlooked (i.e., more type II errors, [26]). Differences in multidimensional structure of zoobenthos were tested with permutational analysis of variance (PERMANOVA, [27]). Differences between the lake parts in their colonization by invertebrates (permanent categorizing factors) were assessed with the use of PERMANOVA (9999 replications, [28]) on a matrix of Bray–Curtis distances. Spatial and temporal variation in community structure, expressed as biodiversity, is a much better measure of the level of community disturbance than species number [29]. The use of the biodiversity index makes it possible to avoid the problem of interpretation of biodiversity of a single sample in respect of the total biodiversity of the lake [30,31]. For this reason, changes in macrozoobenthos diversity were analyzed in selected pars of Lake Łebsko with the use of the Whittaker index (*β*-diversity) according to [32]. Values of *β*-diversity determine variability in species composition as a mean variability among samples and their centering. To detect spatial changes in *β*-diversity, we used one-way ANOVA. Similarity between matrices for animal samples was verified using Bray-Curtis dissimilarity.

To identify which major environmental gradients (physicochemical variables of water, heavy metal concentrations in water and sediments) affect the structure of benthic invertebrate communities, we used canonical correspondence analysis (CCA); [33]. We calculated *p* values using Monte Carlo permutations with Tukey’s modification [33]. The collected data that concentrated on invertebrate groups were log-transformed (log (*n* + 1)), as this was obligatory for the limited unimodal method. Biplots of *t* values indicated which groups of benthic fauna strongly reacted to the studied factors, especially to metals in the lake biotope [34]. Next, using PRIMER 7 software (PRIMER-e, Auckland, New Zealand), we performed BIO-ENV procedure [35], to assess the importance of three categories of variables (physicochemical variables of water, heavy metal concentrations in water and sediments) for explanation of the structure of benthic invertebrate communities. Finally, the basic correlations between all descriptors were investigated independently, using redundancy analysis (RDA).

## 3. Results

### 3.1. Physicochemical Variables of Water

Individual parts of Lake Łebsko differed in most of the studied physicochemical variables (Table 2). Saltwater intrusion into the eastern part resulted in significantly higher values of conductivity, salinity, sulfates (K-W = 10.73–12.91, *p* < 0.0001), and chlorides (K-W = 7.45, *p* = 0.01) than in the western part, which is isolated from such influences. A slow dispersion of brackish seawater entering Lake Łebsko is observed already in its central part (Table 2). Values of conductivity, salinity, sulphates, sodium (Dunn test, *p* < 0.01) and chlorides (*p* < 0.05) were significantly lower there than in the eastern part.

### 3.2. Heavy Metal Analysis

#### 3.2.1. Water

The total concentration of the analyzed heavy metals (Cr, Pb, Ni, Cu, and Al) in water was comparable in the central and western parts (1.116 and 1.113 mg·L^−1^, respectively), and lower in the eastern one, subject to the impact of seawater (0.987 mg·L^−1^). Despite this, concentrations of the heavy metals in water did not differ significantly between lake parts. Cu and Ni reached the highest levels in the central part, while Cr, Al, and Pb in the western one. The ranges of variation of heavy metal concentration in all parts of the lake were the broadest for Al and Pb (Table 3). Mean metal concentrations decreased in the following order: Al > Ni > Pb > Cu > Cr.

#### 3.2.2. Sediments

Total heavy metal concentration decreased in the direction of the eastern part, where it was less than half as high as in the western part (5425 mg·kg^−1^). Each of the analyzed metals significantly differed in concentration in sediments between the lake parts. Markedly higher levels in the western than in the eastern part were recorded for Al and Cu (Dunn’s test, *p* < 0.05), Pb (*p* < 0.01), while Ni and Cr concentrations were significantly higher in the central than in the eastern part (*p* < 0.05). A successive eastward decrease in heavy metal concentrations in sediments (towards the outflow region) concerned only Al and Cu (Table 4). Mean metal concentrations decreased in the following order: Al > Cu > Ni > Pb > Cr.

The western and central parts of Lake Łebsko were characterized by a moderate level of sediment pollution with chromium (CF = 1.1), high levels of nickel (CF = 3.5) and lead (CF = 3.1), and very high contamination with copper (CF = 6.6). In comparison, slightly lower values were recorded in the eastern part (outflow region) for chromium (low), lead (moderate), and copper (high).

PLI values in all parts of the lake exceeded 1 (range 2.7–3.2), indicating that the lake sediments were considerably polluted with the analyzed heavy metals.

### 3.3. Structure of Benthic Invertebrate Communities

In total, 27 taxa of benthic invertebrates were recorded in this study: three polychaetes, four crustaceans, one leech, 11 dipterans, and seven molluscs (62% of them were present in the western part, 44% in the central one, and 60 in the eastern one). In total, 36,043 ind. m^−2^ were collected during the six sampling sessions.

In the western part, on average nearly 800 ind. m^−2^ (of 17 taxa in total) were found during a single sampling session (Table 5). The most abundant were Diptera larvae and Oligochaeta, which jointly accounted for 97.7% of the total catch (61.9% and 35.8%, respectively). Diptera were dominated by *Chironomus* sp., which accounted for 56.1% of the total. Six species (one of chironomid larvae, three gastropods, and two bivalves) were observed only in the western part, but they accounted for only 0.6% of the total catch.

In the central part, only 12 taxa were recorded, with total abundance exceeding 500 ind·m^−2^. The dominance of Diptera larvae increased, whereas *Chironomus* sp. slightly declined (52.4% of the total catch). However, another dipteran, *Polypedilum* sp., and the crustacean *Gammarus duebeni* increased in abundance by 85% and 80%, respectively. Only two species (Gastropoda) were recorded exclusively in the central part of the lake and accounted for 0.6% of the total catch.

The eastern part (permanently connected with the sea) was colonized by 15 taxa, but their abundance was half as high as in the western part. Diptera larvae were still the dominant group but the contribution of *Chironomus* sp. was half as high as in the other parts of the lake (27.1%). They were partly replaced by other members of benthic fauna. Seven species were present only in the eastern part (3 of Crustacea, 1 of Hirudinea, 2 of Chironomidae), and they jointly accounted for 14.3% of the total catch.

Results of PERMANOVA showed considerable changes in univariate descriptors of benthic macrofauna, confirming significant differences between lake parts and sampling sites (Table 6). Results of the statistical analysis showed the existence of differences in animal abundance and α-diversity between the central and eastern parts of the lake (the latter directly connected with the sea by a canal). The analysis of density of the dominant group of invertebrates (Diptera larvae) revealed significant differences between lake parts (mostly eastern vs. western) and sampling sites.

**Figure 2:**
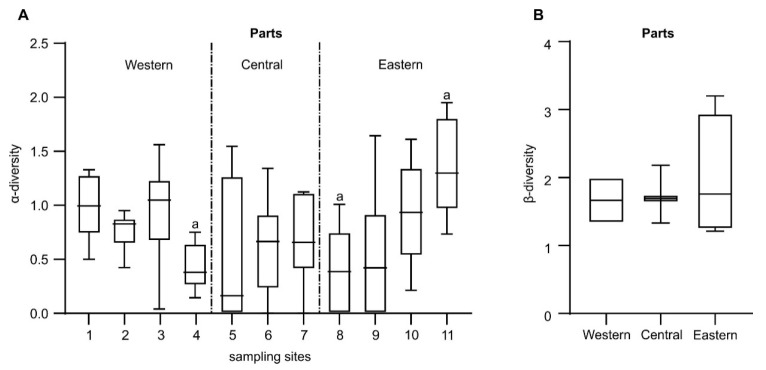
(**A**) Variation of α-diversity index (measured as standard error, SE) at sampling sites (1–11) in different parts of Lake Łebsko and results of one-way ANOVA evaluating differences in results and post hoc test (“a” denotes *p* < 0.05). (**B**) Whittaker index (β-diversity) and its variability (measured as SE) in different parts of the lake.

We compared the distribution of values of α-diversity at individual sites in three lake parts and assessed the significance of differences between sites (Figure 2A). Its values were the highest at the sites affected by saltwater intrusion in the eastern part (9 and 11). Simultaneously, in the same part, extremely low values of this index were recorded at some sites (7 and 8). The lowest mean α-diversity was observed near the mouth of the major tributary (Łeba River) into the lake (4). The Whittaker index (*β*-diversity; Figure 2B) showed a higher variation of macrofauna in the eastern part (connected with the sea) than in the other parts of the lake. In comparison, in the central part, affected by the inflow of river water, most of *β*-diversity values were very similar.

### 3.4. Interactions Between Macrofauna and Environmental Variables

**Figure 3:**
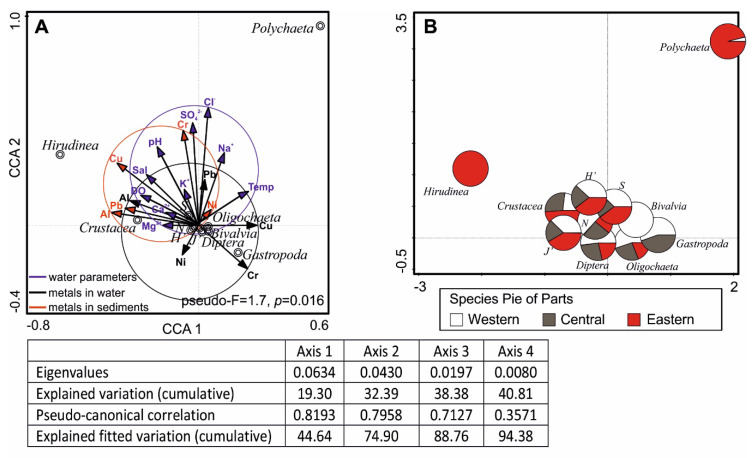
(**A**) Ordination analysis CCA of macrofauna and environmental variables (physico-chemical variables of water, heavy metals in water and sediments) on the principal component 1 and 2; (**B**) pie diagrams based on values of biological descriptors and density of invertebrates groups in parts of lakes. Descriptors: *H*′ = Shannon diversity; *J*′ = Pielou evenness; *S* = number of species; *N* = macrofauna total density; and environmental variables: Temp. = temperature; pH = water reaction; Sal = salinity; DO = dissolved oxygen; Cl^−^ = chlorides; SO_4_^2−^ =sulphates; Na^+^ = sodium; Ca^2+^ = calcium; Mg^2+^ = magnesium.

The analysis of correlation was based on physicochemical variables of water, heavy metal concentrations in water and sediments as well as univariate descriptors of macrofauna and the identified groups of benthic fauna. To a large extent they indicated cumulative effects of environmental factors on invertebrate abundance and diversity (Figure 3A). Major contributors to positive values of the first axis were Cu and Cr concentrations in lake water, and the axis explained 19.9% of the total variance. The second axis was affected mostly by chlorides and sulphates and explained 12.7% of the total variance in benthic fauna. The projection of the abiotic and biotic factors showed that among the identified invertebrate groups, *Gastropoda* abundance was associated with higher values of Cr in water, while Crustacea, with Al concentration in sediments. Physicochemical variables linked with seawater intrusion determined the presence of Polychaeta and partly Hirudinea. This is confirmed by projection of results indicating the preferred lake part (pie procedure, Figure 3B).

Most groups were more abundant in the western part or were found exclusively in this lake part (Bivalvia). Only three groups (Polychaeta, Hirudinea, and Crustacea) reached higher densities in the eastern part. In this context, the central part was the least favourable for benthic invertebrates.

**Figure 4:**
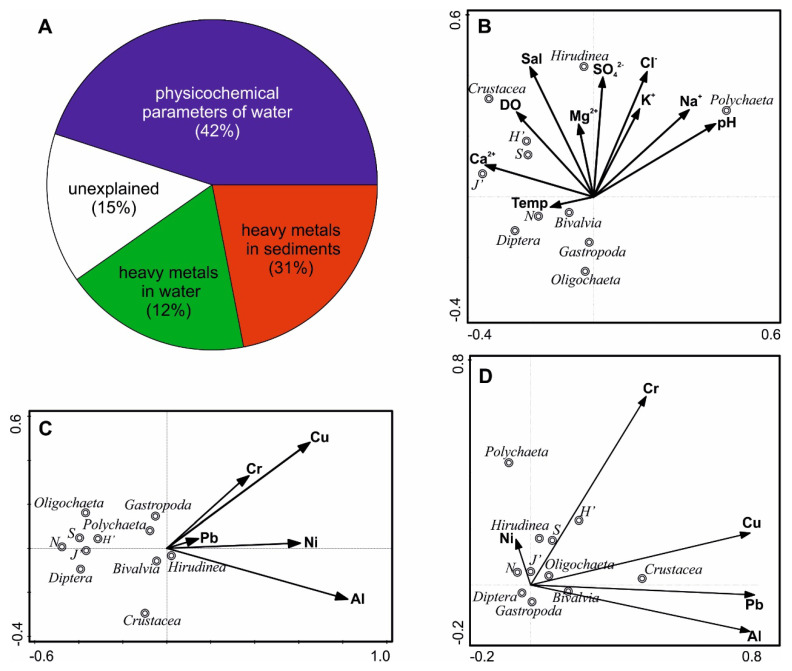
Unique and shared fractions (BIO-ENV procedure) of the total variation of invertebrates composition, explained by physico-chemical variables of water and the contribution of heavy metals in water and sediments (**A**). Results of RDA performed on bottom fauna and environmental data (*p* < 0.05): physico-chemical variables of water (**B**), heavy metals in water (**C**) and sediments (**D**). Descriptors: explanations of the abbreviations as in Figure 3.

BIO-ENV results allowed us to quantify the influence of individual groups of predictors on biological results (Figure 4A). The procedure showed that the physicochemical variables indicating hydrological connectivity with the sea best explained the multidimensional structure of macrofauna (rho = 0.5). The association between benthic fauna and metal concentrations in sediments was slightly weaker (rho = 0.28), while for metal concentrations in water, the relationship was the weakest (rho = 0.11).

RDA projection, taking into account physicochemical and biotic variables of water (Figure 4B) showed that Polychaeta abundance and evenness index values were primarily associated with higher amounts of sodium and calcium, respectively. Simultaneously, the dominant groups of benthic fauna (Oligochaeta and Diptera) as well as molluscs preferred low values of variables linked with seawater intrusion. Levels of heavy metals in water were proportional to the abundance of taxa of benthic fauna and univariate descriptors of this community (Figure 4C). Similar relationships concern the presence of the same metals in sediments, although an apparent positive effect of Ni on Hirudinea abundance is noticeable (Figure 4D).

## 4. Discussion

The major factor determining the structure of benthic communities in lagoons and river mouths is their connectivity with the sea. It allows colonization of estuaries by marine organisms, with the periodical marine dispersal phase [36,37,38]. In Lake Łebsko, benthic macrofauna was composed of typical species of Baltic ICOLLs [6,39,40]. However, near the points of inflow of seawater into the water body, the number of invertebrate species was markedly higher than in freshwater parts of the lake (Table 3). The species collected exclusively at the site with strong saltwater intrusion accounted for a high proportion of the total species number (nearly 20% of identified species). This confirms the hypothesis of high habitat heterogeneity in brackish coastal lakes. The differences concern environmental conditions that affect the structure of benthic animal communities. Similar results were reported earlier [14,41], suggesting an increase in diversity along the salinity gradient, with a simultaneous decrease in zoobenthos abundance. In Lake Łebsko, the westward decline in salinity enabled new freshwater animal species (chiefly molluscs) to colonize the habitat. It is noteworthy that α-diversity was significantly higher in the eastern (most saline) part than in the central part, where fresh water interacts with saltwater most strongly (*p* < 0.01, Figure 3A,B). However, such unstable conditions seem to be preferred by gastropods (*Bithynia tentaculata* and *Theodoxus fluviatilis*). As widely euryhaline species, they are highly plastic, evolutionarily adapted to changes in salinity.

The species colonizing all parts of Lake Łebsko were mostly members of the order Diptera (chiefly chironomid larvae) and Oligochaeta (92% of the total macrofauna abundance). These 2 groups are major components of benthic communities in polymictic, lowland water bodies, including highly eutrophic Baltic coastal lakes [6,42]. Nevertheless, they prefer the lake parts where the contribution of seawater is lower (Figure 3B). In this context, the quantitative change concerns primarily Diptera larvae (mostly of the family Chironomidae), as their contribution to zoobenthos was negatively correlated with increasing salinity gradient (Table 3).

Water circulation in coastal lakes is principally driven by wind, so their salinity is very uneven and changes unpredictably [43]. In spite of permanent hydrological connection with the sea, as in the case of Lake Łebsko, the inflow of brackish Baltic seawater via the narrow channel of the Łeba is small, compared with the lake area [44]. This makes it difficult for oligohaline species colonizing this ecosystem to spread more widely. Sometimes marine species exist in such ecosystems, attesting to their high plasticity in respect of salinity. In fact, our results show that some marine species were present in all parts of the study lake. This was probably due to development of their behavioral strategies (e.g., migrations) to avoid dramatic drops in salinity for a short time. One of such euryhaline species was *Hediste diversicolor* (Polychaeta), which was brought to the estuary by seawater backflow, migrated as far as to the western part of the lake, and survived the gradual decline in salinity. This does not change its preference for predictors characteristic of brackish Baltic waters (i.e., sodium or chlorides, Figure 4B). This species is commonly known to migrate intensively [45,46]. Its distribution pattern is determined by an upper limit of physiological ability to survive in difficult conditions and a lower limit depending on interactions with other species [47].

In available published literature we did not find any information on preferences in respect of the analyzed environmental variables for Mollusca and the dominant groups of zoobenthos: Oligochaeta and Diptera. Among the other invertebrate groups, the presence of Hirudinea depended on high levels of sulphates (Figure 4B). Such an interaction can be explained by symbiosis of Hirudinea with sulfur bacteria [47]. It is supposed that symbiotic α-proteobacteria participate in nitrogen metabolism. According to this concept, our observations do not confirm reports on a lack of influence of physicochemical variables of water (including sulfate concentrations) on Hirudinea communities [48]. The commonly known preference of Crustacea (mostly Gammarus) for highly oxygenated water was confirmed in our study. Nonetheless, this does not modify the general observation that coastal water bodies are characterized by a high specificity of biotopes, which forces aquatic organisms to behave differently than in inland lakes.

There are several reasons why holistic research on ecosystems (taking into account hydrological conditions, water quality, sediment structure and composition, as well as biological components associated with them) is crucial for identifying the rules of their functioning [49]. This is particularly important in the case of ICOLLs, where the combination of marine and terrestrial influences gives rise to dynamic processes [43,50]. In this approach, more and more attention is paid to sediment composition, as it provides integrated and stable knowledge about changes taking place in aquatic ecosystems, as compared with data on changes in lake water. Sediment samples are relatively easy to collect in the field, and heavy metal concentrations in them are at least 100–200-fold higher than in water [51]. Due to this, sediments can be regarded as the most important absorbents of micro- and macropollutants in aquatic ecosystems. Moreover, pollutants (including metal molecules) bound with sediments, can be released to the water column as a result of biological and chemical processes (e.g., adsorption/desorption, redox reactions or decomposition of organisms), as well as physical processes (resuspension), deteriorating the ecological status of water bodies. The absorption of metals by organisms, especially in the benthic zone, depends to a large extent on mobility, concentration, and chemical forms. Thus the understanding of associations between benthic organisms and heavy metal concentrations in various components of the biotope is necessary to assess the structure and dynamics of this animal community [52].

According to our expectations, in this study, Al reached the highest concentrations in both water and sediments, which was linked with the highest abundance of this metal in the lithosphere [53], but also with human activity [54]. It seems that all the heavy metals detected in Lake Łebsko can derive from polluted farmlands, agricultural activity, urbanization, and road traffic, as e.g., Cr is a component of stainless steel and alloy steels, whereas Cu appears in engine lubricants [55]. Higher concentrations of Cu, Ni, and Pb are also associated with economic activity, e.g., Pb is still used in outboard motors [56,57], while most of the biocides that are applied to paint, preserve, and renovate boats, contain Cu [57,58]. The pollution load index (PLI) in all parts of the study lake exceeded 1 (2.7–3.2), attesting to anthropogenic pollution of sediments of Lake Łebsko with the analyzed metals. Thus it can be assumed that the pollution comes from the seaport canal in the town of Łeba as well as the system of drainage ditches surrounding the water body. This is also confirmed by reports of [59,60], suggesting that heavy metals in lake sediments mostly derive from intensive transport of materials from the catchment area into the lake or are leached from geological deposits. Despite this, in Lake Łebsko, concentrations of the analyzed metals did not exceed the maximum levels allowed in Polish waters of the highest water quality class [61]. This can be explained by the location of the lake in an area protected by law, limiting the possibility of areal loading from its direct catchment [17].

This fact affects the structure of invertebrate communities colonizing the lake bottom. In Lake Łebsko, the dominant groups are chironomid larvae (Diptera) and oligochaetes (see Table 2). The Chironomidae have been used for a long time as perfect bioindicators in Baltic coastal lakes [6,38,39]. Their ecological success is a consequence of evolutionary and physiological adaptive abilities, which enable them to live in extreme conditions. Survival of their larvae in the environment depends on variability of environmental factors, including water temperature, pH, and dissolved oxygen [62,63,64]. According to [65], sensitive and moderately pollution-tolerant species disappear and only the highly tolerant ones can survive the deterioration of environmental conditions. As a consequence, in ecosystems strongly loaded with heavy metals, chironomids are major components of the bottom fauna. In degraded aquatic ecosystems they are accompanied by oligochaetes, which are also regarded as a group highly resistant to all kinds of pollution, including salinity [66]. The dominance of Oligochaeta in polluted waters is confirmed by studies of [65,67] on the Yangtze River and Lake Taihu, respectively, showing that the Oligochaeta are highly tolerant organisms and are preferred indicators for assessment of unfavorable effects of heavy metal pollution in aquatic ecosystems. For both groups of organisms, it is important that they are able to excrete heavy metals or retain them in tissues also in function of their larval development [68].

Macroinvertebrate communities living in water bodies polluted with heavy metals are characterized by lower abundance and/or biomass [69,70]. Our findings confirm earlier reports that there is a clear causal relationship between metal concentrations and diversity of benthic communities. This applies especially to sensitive taxa (molluscs, crustaceans), as evidenced by our results (Table 5). Sediments containing micronutrients (heavy metals) can inhibit or stimulate the growth of macroinvertebrate populations, but simultaneously the activity of invertebrates determines sediment properties [71].

Our analyses indicate that in Lake Łebsko the major predictor responsible for the observed qualitative and quantitative structure of benthic communities is the hydrological connectivity and the associated intrusion of seawater. Our results (Figure 4A) indicate that physicochemical variables of water, associated with the hydrological connection of the lake with the sea, best explained the multidimensional structure of macrofauna. It can be assumed that permanent seawater intrusion has a refreshing influence on the water body and increases the heterogeneity of habitats, which can be colonized by less abundant but more diverse benthic animal communities (Table 6). Simultaneously, the western part (mostly affected by waters from the catchment area) provided favorable conditions for a high abundance of only two groups: oligochaetes and chironomid larvae. As a consequence, greater abundance and low biodiversity were observed in the western part, while low abundance and the highest diversity, in the eastern part. The combination of marine and terrestrial factors in the central part caused strong changes within the biotope, unfavorable for invertebrate development. This is reflected in the lowest values of density and α-diversity, as compared with other parts of the lake. This finding is consistent with [6], who reported that intrusion of seawater into transitional lakes forces benthic fauna to adapt to new conditions, which lead to a decrease in animal abundance and diversity, in comparison with areas with stable conditions.

## 5. Conclusions

In conclusion, knowledge about metal concentrations in water and sediments cannot be used as the only criterion of habitat health assessment or potential toxicity. In natural waters, metals are subject to interactions of their various forms and this leads to their separation into individual subsystems. This is most particularly conspicuous in studies of ICOLLs, where the impact of the sea is the major predictor, initiating nearly all biological and geochemical changes. Simultaneously, determination of the hierarchy of abiotic factors is necessary for identification of the mechanisms of functioning of biological communities in coastal water bodies according to the evolutionary of ecosystem paradigm. Only in this way, we can develop proper procedures for management of the shoreline.

## Figures and Tables

**Table 1:** Morphometric characteristics and classification of Lake Łebsko. Water body type corresponds to its hydrological connectivity (water exchange with the sea) and level of salinity [6,18].

Geographic Coordinates	Area (ha)	Mean Depth (m)	Capacity (10^6^ m^3^)	Level of Salinity	Hydrological Connectivity	SB	Type of Habitat
54°43′ N, 17°25′ E	7020	1.6	113.5	β-oligohalinity	Permanently connected, seawater enters it by canal of Łeba River	276	Brackish

SB = average number of days of seawater backflow in 2000–2015 [18].

**Table 2:** Statistical summary of physicochemical variables of water in 2014–2015 in the western (inflow), central, and eastern (outflow) parts of Lake Łebsko with ANOVA results.

Statistics	T	pH	EC	DO	Sal	Cl^−^	SO_4_^2-^	Na^+^	Mg^2+^	Ca^2+^	K^+^
	°C	-	μS cm^−1^	%	PSU	mg·L^−1^	mg·L^−1^	mg·L^−1^	mg·L^−1^	mg·L^−1^	mg·L^−1^
Western part (inter region)											
Min	12.8	8.18	2980	62.2	1.57	830.3	103.2	30.5	2.0	33.2	5.0
Max	26.1	8.86	6336	126.8	3.49	1511.9	225.7	881.2	105.3	96.2	34.3
Mean	16.0	8.50	4652	88.8	2.50 *	1216.5*	178.4*	633.4*	69.9	50.3	24.3
Central part											
Min	10.5	8.10	1747	54.0	0.93	944.1	118.7	539.9	52.2	33.2	16.6
Max	25.9	8.80	6395	114.3	4.32	1707.9	254.1	1099.1	116.8	97.8	42.4
Mean	15.6	8.42	5421	89.1	2.95	1363.3	194.9	748.9	82.6	53.0	28.2
Eastern part (outer region)											
Min	11.3	7.54	3132	29.0	1.64	1099.0	163.2	28.4	2.0	32.1	4.5
Max	25.2	8.61	14615	124.9	8.53	6777.2	630.1	2270.1	279.6	98.9	83.2
Mean	16.0	8.18	7626	92.2	4.23*	2166.4*	284.8*	980.1*	98.3	54.7	32.5
ANOVA (*p*)	0.58	**0.00**	**0.00**	0.62	**0.00**	**0.01**	**0.00**	**0.01**	0.06	0.71	**0.05**

Environmental predictors: T = temperature; EC = conductivity; Sal = salinity; DO = dissolved oxygen, Cl^-^ = chlorides; SO_4_^2−^ =sulphates; Na^+^ = sodium; Ca^2+^ = calcium; Mg^2+^ = magnesium; *p* values modified by the Tukey procedure for multiple comparisons show no significant effect; (*p* < 0.05); *– statistically significant differences between parts, Dunn’s test, *p* < 0.05; bold values indicate significant differences.

**Table 3:** Concentrations of major heavy metals in water (mg·L^−1^) in 2014–2015 in the western (inflow), central, and eastern (outflow) parts of Lake Łebsko with ANOVA results.

Statistics	Al	Cr	Ni	Pb	Cu
Western part (inflow region)					
Min	0.161	0.002	0.007	0.003	0.006
Max	2.974	0.057	0.078	0.349	0.040
Mean	0.995	0.018	0.034	0.049	0.017
Central part					
Min	0.115	0.002	0.006	0.003	0.008
Max	3.928	0.046	0.147	0.159	0.058
Mean	0.943	0.016	0.090	0.040	0.027
Eastern part (outflow region)					
Min	0.125	0.001	0.005	0.002	0.010
Max	4.234	0.034	0.060	0.088	0.037
Mean	0.864	0.014	0.055	0.035	0.019
ANOVA (*p*)	0.49	0.30	0.76	0.88	0.58

*p* values modified by the Tukey procedure for multiple comparisons show no significant effect.

**Table 4:** Concentrations of major elements in sediments (mg·kg^−1^) in 2014–2015 in the western (inflow), central, and eastern (outflow) parts of Lake Łebsko with ANOVA results.

Statistics	Al	Cr	Ni	Pb	Cu
Western part (inflow region)					
Min	176.26	10.30	8.34	2.15	3.40
Max	9946.06	61.62	86.44	91.73	94.32
Mean	5283.28 *	28.64	36.17	30.29 *	46.52 *
Central part					
Min	412.26	0.48	13.72	9.48	6.69
Max	10216.40	51.81	184.43	99.84	84.83
Mean	4692.69	30.48 *	44.85*	33.50	44.19
Eastern part (outflow region)					
Min	238.48	10.52	5.95	2.22	11.52
Max	6010.93	91.53	76.36	225.53	63.63
Mean	2595.96 *	26.98*	33.02 *	29.31 *	36.90 *
ANOVA (*p*)	**0.01**	**0.00**	**0.05**	**0.00**	**0.05**

*p* values modified by the Tukey procedure for multiple comparisons show no significant effect; *—statistically significant differences between parts, Dunn’s test, *p* < 0.05; bold values indicate significant differences.

**Table 5:** Composition of macrozoobenthos (ind·m^−2^) at the different sampling sites (1–11) in parts of Lake Łebsko in 2014–2015, and results of one-way ANOVA evaluating differences between the parts.

Taxa and Indices	Western				Central			Eastern				*p*
	1	2	3	4	5	6	7	8	9	10	11	
1-OLIGOCHAETA	1096.3	3866.6	1111.1	800.0	592.5	488.9	2133.4	992.6	429.5	518.7	415.5	**0.041**
2-POLYCHAETA	44.4								29.6	14.8	177.8	0.191
*Hediste diversicoilor*	44.4										163.0	
*Pygospio elegans*												
*Mysis mixta*									29.6	14.8	14.8	
3-CRUSTACEA	14.8	102.9	103.7	44.4	74.0	251.8			178.1	177.7	148.1	0.506
*Asellusa quaticus*									74.1		44.4	
*Gammarus duebeni*		14.1	74.1		74.0	251.8			59.6	133.3	88.9	
*Gammarus oceanicus*									44.4	14.8	14.8	
*Neomysis integer*	14.8	88.8	29.6	44.4						29.6	-	
4-HIRUDINEA											14.8	0.170
*Pisicola geometra*												
5-DIPTERA LARVAE	918.4	3733.2	1688.7	5526.0	1436.9	3229.5	1288.8	755.5	1703.7	1644.5	133.3	**0.019**
*Chironomus* sp.	399.9	3525.9	1348.1	5466.8	1140.7	2977.7	888.9	651.9	1392.6	325.9	14.8	
Chironomidae n.det.	29.6	29.6		59.2	14.8	29.6	14.8	14.8		88.9		
*Dicrochironomus* sp.							29.6			29.6	14.8	
*Procladius* sp.		14.8	222.2		222.2	14.8	14.8					
*Polypedilum* sp.		14.8			29.6	133.3						
*Psectrocladius* sp.								14.8		14.8		
*Bezzia* sp.	29.6	148.1	118.4		29.6	74.1	44.4					
*Microtendipes* sp.										74.1		
*Sergentia* sp.	414.9						281.5			948.2	103.7	
*Einfeldia* sp.	29.6						14.8	74.0	311.1	163.0		
*Clunio* sp.	14.8											
6-MOLLUSCA		14.8	74.0	14.8	29.6		29.6					
A-GASTROPODA		14.8	44.4	0.0	29.6		29.6					0.280
*Bithynia tentaculata*							29.6					
*Valvata piscinalis*		14.8										
*Theodoxus fluviatilis*					29.6							
*Potamopyrgus jenkinsi*			14.8									
*Hydrobia ulvae*			29.6									
B-BIVALVIA			29.6	14.8								0.511
*Dreissena polymorpha*				14.8								
*Anodonta anatina*			29.6									
*N*, ind. m^−2^	345.7	1286.3	496.3	1064.2	355.5	661.7	575.3	291.4	390.2	392.6	148.3	
*S*, species m^−2^	9	9	9	5	8	7	9	5	7	12	10	
*N*, ind. m^−2^ per part	798.1				530.8			366.7				**0.018**
*S*, species m^−2^ per part	17				12			15				0.310
α-diversity (*H*’)	0.781				0.606			0.804				**0.011**
Evenness (*J*’)	0.654				0.477			0.717				0.411

*p* values modified by the Tukey procedure for multiple comparisons show no significant effect; bold values indicate significant differences.

**Table 6:** Permutational analysis of variance (PERMANOVA) results, testing the effects of three parts of Lake Łebsko (western, central, and eastern) and sampling sites on the total density, diversity of invertebrates, and density of Diptera larvae. Analysis based on Bray-Curtis dissimilarity indices.

Variance	Sources of Variation	df	SS	MS	Pseudo *F* Values	*p* (MC)
Density	Parts	2	2267984	113399	4.276	**0.018**
	Sites	10	1365870	236541	3.145	**0.021**
	Residual	63	1670603	265175		
Pair-wise tests						
Compared of parts						
West vs Central					2.75	0.23
Central vs East					18.10	**0.007**
West vs East					7.66	0.57
Total α-diversity	Parts	2	0.620	3.1000	4.806	**0.01**
	Sites	10	0.555	2.9574	3.587	**0.02**
	Residual	63	4.064	0.6454		
Pair-wise tests						
Compared of parts						
West vs Central					243.40	0.07
Central vs East					482.20	**0.01**
West vs East					206.80	0.67
Diptera density	Parts	2	0.0098	0.0049	0.0781	**0.02**
	Sites	10	0.0147	0.0258	0.1487	**0.001**
	Residual	63	4.4960	0.0624		
Pair-wise tests						
Compared of parts						
West vs Central					264.60	**0.01**
Central vs East					320.80	**0.004**
West vs East					669.90	**0.03**

*p* (MC): *p*-value of Monte Carlo permutation test; bold values indicate significant differences at *p* < 0.05.
